# Roles of Psychological Flexibility, Parenting Competence, and Asthma Management Self-Efficacy in the Functioning Outcomes of Parents of Children with Asthma Co-Occurring with Attention-Deficit/Hyperactivity Disorder

**DOI:** 10.3390/ejihpe14110186

**Published:** 2024-10-29

**Authors:** Yuen Yu Chong, Pui Tik Yau, Joycelyn Yee Man Kwan, Wai Tong Chien

**Affiliations:** The Nethersole School of Nursing, Faculty of Medicine, The Chinese University of Hong Kong, Hong Kong SAR, China; jamyau@cuhk.edu.hk (P.T.Y.); joycelynkwan@cuhk.edu.hk (J.Y.M.K.); wtchien@cuhk.edu.hk (W.T.C.)

**Keywords:** psychological flexibility, ADHD, asthma, family, parenting competence

## Abstract

Asthma and ADHD represent prevalent pediatric conditions, with the former being a physical disorder and the latter being a neurodevelopmental disorder. This study examined the influence of parental psychological flexibility (PF)—the ability to adapt to evolving situational demands, shift perspectives, and balance competing priorities—alongside parenting competence and asthma management self-efficacy on family functioning and parental psychological adjustment in families with children exhibiting concurrent asthma and ADHD symptoms. Baseline data were analyzed from 130 parents (mean age = 40.3 years, SD = 5.5; 88.9% mothers) of children diagnosed with both asthma and ADHD (mean age = 8.0 years, SD = 2.2; 74.6% boys), who were participating in a randomized controlled trial evaluating an Acceptance and Commitment Therapy (ACT)-based parenting intervention. An adjusted structural equation model revealed that greater parental psychological inflexibility was significantly associated with poorer family functioning (β = −0.61, 95% CI [−0.74, −0.33], *p* < 0.001) and increased psychological maladjustment (β = 0.48, 95% CI [0.32, 0.63], *p* < 0.001), accounting for intercorrelations with parenting competence and parental asthma management self-efficacy. Additionally, parental psychological flexibility was found to mediate the relationship between parenting competence and both family functioning and psychological adjustment. These findings underscore the importance of targeting parental psychological inflexibility and enhancing parenting competence in interventions to improve family dynamics and parental mental health and thereby suggest a shift from the traditional focus on self-efficacy in symptom management for pediatric asthma and ADHD.

## 1. Introduction

Asthma affects approximately 10% of the global pediatric population, while attention-deficit hyperactivity disorder (ADHD) prevalence is around 10% in regions like the United States and China [1,2,3]. However, these rates can vary depending on cultural, geographical, and age-related factors [4]. For instance, globalization can lead to increased exposure to pollutants and allergens [5], while climate change is associated with rising temperatures and altered weather patterns, potentially exacerbating respiratory conditions like asthma [6]. Understanding how asthma and ADHD prevalence and interaction vary across different age groups is crucial, as these conditions may manifest differently at various developmental stages [7]. Ecological modeling, which accounts for age-related variations, could provide valuable insights into the dynamics between these conditions and help identify critical periods for intervention [8].

Asthma and ADHD impose significant challenges on affected children and their families. Asthma can limit physical activities due to recurrent wheezing attacks [9], while ADHD is associated with academic challenges, social relationship strains, and risk-taking behaviors [10]. Notably, the prevalence of asthma is higher among children with ADHD, estimated at 36.6%, compared to 24.3% in their peers who do not have ADHD [11], pointing toward a potential shared etiological pathway. In the literature, several hypotheses have been proposed to explain this comorbidity. One hypothesis involves neuro-immunological interactions, where chronic inflammatory processes associated with asthma could impact neurological development, contributing to ADHD symptoms [12]. Sleep disturbances are another critical factor linking asthma and ADHD. Asthma-related sleep disturbances, such as nocturnal wheezing, can exacerbate ADHD symptoms by disrupting sleep and impairing attention and behavior [13]. Conversely, ADHD-related insomnia, driven by hyperarousal, may worsen asthma by increasing night-time awakenings and reducing overall sleep quality, potentially heightening airway inflammation [14]. Furthermore, asthma has also been postulated as an early risk indicator for ADHD, with a diagnosis of asthma in early childhood (ages 0–3) associated with a higher likelihood of subsequent ADHD development [15,16]. This association persists after adjusting for maternal age at birth, socioeconomic status, and child sex [17]. A twin study supports this link, showing that children with asthma are nearly twice as likely to have ADHD [18]. Children with both conditions also experience more frequent asthma attacks, suggesting a potential synergistic interaction, where asthma and ADHD symptoms exacerbate each other [19]. This interaction could be due to the stress and anxiety associated with managing one chronic condition, which may worsen the symptoms of the other [20].

Parents caring for a child with concurrent diagnoses of asthma and ADHD experience a distinctive and heightened caregiving burden [21,22]. The symptoms of ADHD, such as impulsivity and hyperactivity, may hinder adherence to asthma management regimens, increase exposure to asthma triggers, and complicate the accurate assessment of asthma symptoms [23]. Furthermore, inattention may lead to the inadequate use of preventative medications, culminating in poorly controlled asthma and a higher frequency of hospital visits [23]. The complexity involved in managing a child’s asthma in conjunction with ADHD symptoms can affect parental psychological adjustment, manifesting in maladaptive coping strategies that range from avoidance to over-vigilance. Such coping responses may, in turn, heighten parents’ vulnerability to mental health conditions, notably, anxiety and depression [24]. Moreover, the persistent stress and disruptions linked to managing these co-existing conditions can strain family dynamics, creating tension in interpersonal relationships and potentially diminishing overall family functioning [25].

### 1.1. Conceptual Frameworks

Family Systems Theory provides a comprehensive lens for conceptualizing how stressors, such as managing a child’s dual chronic conditions (e.g., asthma and ADHD), impact the entire family unit. Families are understood as interconnected subsystems, including parent–child and coparent relationships [26]. When caregiving demands increase, stress diffuses across these subsystems, disrupting the family’s homeostatic balance. This process aligns with the spillover hypothesis [27], which posits that stress in one domain (e.g., caregiving) can spill over into other areas, such as marital conflict or parent–child interactions, ultimately affecting overall family functioning [28]. The Family Stress Model further elaborates on how external stressors—such as healthcare demands, financial pressures, and the emotional burden of caregiving—can disrupt parental psychological adjustment, which in turn negatively impacts child well-being [29].

While Family Systems Theory and the Family Stress Model offer broader frameworks for understanding the interconnectedness of family members (e.g., parents and children), it is essential to explore specific modifiable factors that can enhance psychological adjustment and family functioning. In this study, we propose the concurrent examination of self-efficacy, parenting competence, and psychological flexibility, as these factors have been identified as key psychological resources for fostering family resilience in caregiving contexts. Social Cognitive Theory (SCT) provides a foundation for understanding parental self-efficacy [30], which refers to a parent’s belief in their ability to manage their child’s chronic conditions effectively [31,32]. High levels of self-efficacy not only contribute to better asthma control through tasks such as medication adherence and symptom monitoring, they also enhance parental psychological adjustment, ultimately improving overall family well-being [31,32]. Similarly, parenting competence, defined as the ability to skillfully manage caregiving tasks (e.g., asthma action plans, handling acute episodes), plays a critical role in reducing parental stress and promoting family stability [33]. Competence in caregiving mitigates the chaos often associated with pediatric health issues, creating a more predictable and orderly family environment that supports better family functioning and parental adjustment [34]. Additionally, psychological flexibility, rooted in Acceptance and Commitment Therapy (ACT) and Relational Frame Theory (RFT) [35], empowers parents to remain open to difficult emotions and engage in value-driven behaviors despite adversity [36,37]. Studies have consistently demonstrated the association between psychological flexibility and positive outcomes in parental psychological health, particularly among parents of children with neurodevelopmental and chronic conditions [38,39,40,41]. Thus, integrating concepts from SCT and ACT leads to a targeted framework for identifying modifiable factors that may improve parental well-being and family functioning.

Indeed, psychological flexibility, parenting competence, and parental self-efficacy are possibility related, as each construct may reinforce the others in a reciprocal manner. For example, psychological flexibility may enhance parenting competence by enabling parents to remain open to new strategies and approaches in managing their child’s condition and thereby improve their caregiving efficacy [42]. As parents become more competent and adaptable in their caregiving, their confidence in managing their child’s asthma effectively—i.e., their parental self-efficacy—may also increase [43]. Conversely, higher levels of parental self-efficacy can reduce caregiving-related anxiety and stress, thereby promoting greater psychological flexibility and emotional resilience [36]. Despite the importance of these constructs, to date, no studies have concurrently examined how all three inter-related factors—self-efficacy, parenting competence, and psychological flexibility—contribute to parental psychological adjustment and family functioning. Moreover, few studies have focused on the parents of children with asthma co-occurring with ADHD, a population facing unique caregiving challenges. These parents must manage the physical health demands of asthma while also navigating the behavioral and emotional difficulties associated with ADHD, making this population distinct yet underserved and in need of targeted examination.

### 1.2. The Current Study

Building on the theoretical frameworks arising from Family Systems Theory and the Family Stress Model [26,29], this study aimed to investigate whether psychological flexibility, parenting competence, and asthma management self-efficacy were simultaneously associated with psychological adjustment and family functioning and whether psychological flexibility mediated these associations among Hong Kong Chinese parents of children diagnosed with asthma and co-occurring ADHD. We hypothesized that greater psychological inflexibility would be associated with poorer psychological adjustment and family functioning. In contrast, higher levels of parenting competence and asthma management self-efficacy were expected to be associated with better psychological adjustment and family functioning. Additionally, we hypothesized that psychological flexibility would mediate the relationship between parenting competence and asthma management self-efficacy (as independent variables) and psychological adjustment and family functioning (as dependent variables) among Hong Kong Chinese parents of children with asthma and co-occurring ADHD. By exploring these inter-relationships, our study could contribute to the growing body of literature on family functioning in the context of dual chronic conditions and provide empirical support for interventions aimed at enhancing parental psychological resources to improve overall family well-being.

## 2. Materials and Methods

### 2.1. Participants and Procedures

This cross-sectional study utilizes baseline data from an ongoing clinical trial (ClinicalTrials.gov: NCT04991649) that examines the efficacy of an Acceptance and Commitment Therapy (ACT)-based asthma management program for parents of children with comorbid asthma and ADHD, assessing health outcomes over a 12-month follow-up period. The COVID-19 pandemic necessitated quarantine and social distancing measures, which led to a significant reduction in clinic attendance, dropping to 30% of the usual rate. This decrease in patient visits directly impacted our ability to recruit participants and collect data, necessitating an extension of the recruitment and data collection period to 18 months. Hence, the clinical trial recruited participants from August 2021 to February 2023 at two pediatric outpatient clinics in the Department of Paediatric and Adolescent Medicine at a regional hospital in Hong Kong. This hospital provides services to three districts in Hong Kong, which collectively represent 16.2% of the Hong Kong child population under the age of 14 [44]. Prior to initiating the study, ethical approval was secured from both the hospital and university review boards.

The eligibility criteria for parent–child dyads included parents aged between 18 and 65 years, who were primary caregivers, living with the child, fluent in Cantonese, and contactable by telephone. The child was required to be aged 3–12 years, with a physician-confirmed diagnosis of asthma (ICD–10 codes J45, J46), a Childhood Asthma Control Test (C-ACT) score of ≤19 (indicating poorly controlled asthma) [45], and a concurrent diagnosis of ADHD according to DSM-5 or ICD-10 criteria as documented in the child’s clinical record. The exclusion criteria encompassed participation in another asthma-related intervention study or the presence of significant medical conditions in the child, such as congenital issues, dependency on oxygen, or a tracheostomy.

A consecutive sampling strategy was employed. Upon the attendance of children who met the eligibility criteria at the clinics for asthma-related assessments and treatments, the accompanying parent was screened for eligibility by a trained research assistant. Written informed consent was obtained from eligible parents, who were subsequently invited by nursing staff to complete a self-administered structured questionnaire in a private consultation room, to collect clinical information pertaining to both the parent and child.

### 2.2. Measures

Psychological flexibility. The 7-item Acceptance and Action Questionnaire-II (AAQ-II) was employed to assess the inverse of psychological flexibility in parents, focusing on the extent to which internal experiences interfere with value-orientated actions and present-moment awareness [46]. Respondents rated items on a 7-point Likert scale, ranging from 1 (never true) to 7 (always true). An example item is, “Painful experiences and memories make it difficult for me to live a meaningful life”. Higher aggregate scores correspond to increased psychological inflexibility. The AAQ-II has demonstrated strong internal consistency (α = 0.88) and reliable test–retest measures (r = 0.79–0.81) in a sample of Hong Kong parents [47].

Asthma management self-efficacy. The 13-item Parent Asthma Management Self-Efficacy scale (PAMSE) was used to evaluate parents’ confidence in preventing and managing acute asthma exacerbations in their children [32]. This scale consists of two subscales and uses a 5-point Likert scale, ranging from 1 (not at all sure) to 5 (completely sure). An illustrative question is, “How sure are you of your child taking their medication?” Higher scores on the total scale or subscales indicate better asthma management capabilities. The PAMSE has shown adequate internal consistency (α = 0.77–0.82) and good test–retest reliability (ICC = 0.76–0.87) among Hong Kong parents [47].

Parenting competence. To evaluate parents’ self-perceived competency in handling parenting responsibilities, the 17-item Parenting Sense of Competency (PSOC) scale was utilized [48]. Responses were recorded on a 6-point Likert scale, ranging from 1 (strongly disagree) to 6 (strongly agree). A sample item includes, “I think I can be a role model for people who just became parents and show them how to be good parents”. Higher total scores on the PSOC reflect greater perceived parenting competence. The Chinese version of the PSOC has demonstrated satisfactory internal consistency (α = 0.77–0.85) and strong test–retest reliability (r = 0.87) among Hong Kong parents [49].

Parental psychological adjustment. The 25-item Parent Experience of Child Illness (PECI) scale was administered to assess psychological adjustment in parents caring for a child with chronic illnesses [50]. This scale uses a 5-point Likert scale, ranging from 0 (never) to 4 (always), and includes two subscales: PECI Distress (covering guilt, worry, unresolved sorrow, anger, and long-term uncertainty) and PECI Resources (emotional resources). For this study, scores from the PECI Distress subscale were used to indicate levels of psychological adjustment. An example item is, “I worry about my child’s future”, with higher scores indicating greater psychological maladjustment. The PECI has shown adequate internal consistency (α = 0.72–0.89) and test–retest reliability among Hong Kong parents (r = 0.83–0.86) [47].

Family functioning. The impact of comorbid asthma and ADHD on family functioning and health-related quality of life was assessed using the 36-item Pediatric Quality of Life Inventory Family Impact Module (PedsQL FIM) [51]. This module includes 8 subscales: physical functioning (6 items), emotional functioning (5 items), social functioning (4 items), cognitive functioning (5 items), communication (3 items), worry (5 items), daily activities (3 items), and family relationships (5 items). Responses are recorded on a 5-point Likert scale, ranging from 0 (never) to 5 (always), and items are then converted to a 0–100 scale (0 = 100, 1 = 75, 2 = 50, 3 = 25, 4 = 0), with higher scores reflecting better functioning. Example statements include, “I feel tired during the day,” and “I find it hard to receive support from others.” The PedsQL FIM has demonstrated excellent internal consistency (α = 0.96) in studies involving parents of children with asthma and heart disease in Mainland China [52].

### 2.3. Data Analysis

Descriptive statistics and correlational analyses of the observed variables were performed using SPSS version 29.0. Multivariate normality was verified via Mahalanobis distance, with significance confirmed at the *p* < 0.001 threshold, and no outliers were identified. Although the outcome variables exhibited mild deviations from normality, with kurtosis values ranging from 0.34 to 0.76, they were deemed suitable for further analysis.

Given the complexity of the relationships among the variables in our study, we employed Structural Equation Modeling (SEM) as the primary analysis technique, using maximum likelihood estimation via SPSS AMOS version 29.0 (IBM Corp., Chicago, IL, USA). SEM was chosen because it allows for the simultaneous examination of multiple observed variables—specifically, psychological inflexibility (AAQ-II total score), asthma management self-efficacy (PAMSE mean score), and parenting competence (PSOC total score)—and their relationships with latent variables, namely, family functioning (PedsQL FIM subscale scores) and parental psychological maladjustment (PECI distress subscale scores). Unlike traditional regression models, SEM can account for the inter-relationships and covariances between these observed variables, providing a more comprehensive understanding of the underlying mechanisms.

Initially, measurement models for the two latent variables were developed to confirm whether the constructs exhibited significant factor loadings. Following this, two structural models were employed to test the hypothesized relationships between the specified observed and latent variables, assessing the model’s fit using the following indices: Comparative Fit Index (CFI) ≥ 0.90; Tucker–Lewis Index (TLI) ≥ 0.90; standardized root mean square residual (SRMR) ≤ 0.10; and root mean square error of approximation (RMSEA) ≤ 0.08, which together indicate an acceptable model fit [53]. The SEM was adjusted for various sociodemographic variables, including the parents’ age and relationship to the child, the child’s age and sex, and the current use of inhaled corticosteroids and CNS and/or non-CNS stimulants for managing asthma and ADHD symptoms. Insignificant confounders, along with their associated paths with the latent constructs, were subsequently removed to refine the model. The effect sizes for absolute correlation (r) were classified as small (>0.10), medium (>0.30), or large (>0.50) [54]. All statistical tests were two-sided, with a *p*-value < 0.05 considered indicative of statistical significance.

## 3. Results

### Characteristics of the Parent–Child Dyads

A total of 130 parent–child dyads participated in the study. The parents, predominantly mothers (88.9%), with over half identifying as homemakers (54.6%), had a mean age of 40.3 years (SD = 5.54). The children, primarily boys (74.6%), had a mean age of 8.00 years (SD = 2.22). The majority of parents reported having completed education up to the secondary school level (66.2%). On average, the children were diagnosed with asthma at 3.56 years of age (SD = 2.09), and 37.7% (*n* = 49) required daily inhaled corticosteroids for asthma management. Over the past year, approximately one-third of the children (n = 38) experienced at least one unscheduled medical visit due to asthma exacerbation, and 11 children necessitated hospitalization. All children had a comorbid ADHD diagnosis as per DSM-5 criteria, with 53.8% currently receiving child rehabilitation services and less than one-fifth (n = 21) being on CNS and/or non-CNS stimulants for symptom management. Additionally, a subset of the sample had other neurodevelopmental conditions: autism spectrum disorder (23.1%, n = 30), developmental language disorder (16.2%, n = 21), and dyslexia (13.8%, n = 18).

Table 1 presents the zero-order correlations between the observed variables. Intercorrelations between the observed variables among the latent variables, including psychological adjustment measured by the PECI subscale scores (r ranged from 0.60 to 0.71, *p* < 0.001) and family functioning measured by the FIM subscale scores (r ranged from 0.37 to 0.64, *p* < 0.001) were all significant with medium-to-large effect sizes. Notably, the AAQ-II score was significantly correlated with both PECI subscale scores (r ranged from 0.42 to 0.55, *p* < 0.001) and FIM subscale scores (r ranged from −0.63 to −0.39, *p* < 0.001). The strength of these correlations exceeded those observed with the PAMSE and PSOC scores, suggesting that the relationship between psychological inflexibility and psychological maladjustment, as well as family functioning, is more pronounced than that with asthma management self-efficacy and parenting competence. The results of confirmatory factor analysis are presented in Table A1. Standardized factor loadings for all the observed variables were significant (all *p* < 0.001) with factor loadings ranging between 0.71 and 0.85.

**Figure 1:**
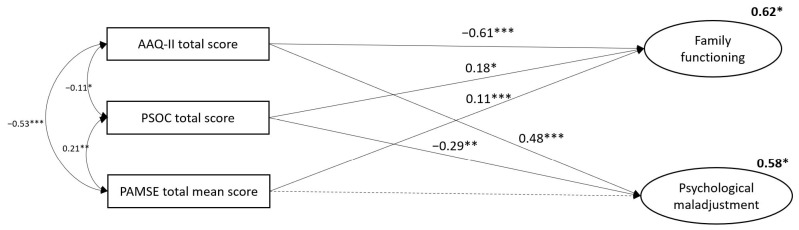
The final structural equation model. Note. Latent variables are presented as ellipses and observed variables are presented as rectangles. Solid lines illustrate statistically significant relationships, and dashed lines illustrate those that are not. Squared multiple correlation coefficients, showing the variance that each variable explains, are placed in the upper right corner of each variable’s representation. The model was adjusted for the following sociodemographic variables: parents’ age and relationship with the child, the child’s age and sex, and the current use of inhaled corticosteroids and CNS and/or non-CNS stimulants for asthma and ADHD symptom management. Insignificant confounders and their associated paths were subsequently removed to streamline the model. AAQ-II, Acceptance and Action Questionnaire-II; PSOC, Parenting Sense of Competency; PAMSE, Parents’ Asthma Management Self-Efficacy; FIM, Pediatric Quality of Life Inventory Family Impact Module; and PECI, Parent Experience of Child Illness. Significance is indicated with * *p* < 0.05, ** *p* < 0.01, and *** *p* < 0.001.

Figure 1 shows the full trimmed SEM adjusted for the current use of CNS and/or non-CNS stimulant. Goodness-of-fit indices for the SEM yielded a good model fit (χ^2^/df = 166.41/81 = 2.05, RMSEA = 0.08, CFI = 0.90, TLI = 0.91, SRMR = 0.07). The Bollen–Stine bootstrap test yielded a *p*-value of 0.09, which is not statistically significant. Bootstrap analyses revealed bias values ranging from 0.001 to 0.005 across parameter estimates, suggesting negligible divergence from the original maximum likelihood estimates. Psychological inflexibility was found to be significantly associated with family functioning (standardized beta coefficient, β = −0.61, 95% CI [−0.74, −0.33], *p* = < 0.001) and psychological maladjustment (β = 0.48, 95% CI [0.32, 0.63], *p* = < 0.001). In parallel, parenting competence showed significant associations with family functioning (β = 0.18, 95% CI [0.04, 0.31], *p* = 0.013) and psychological adjustment (β = −0.29, 95% CI [−0.46, −0.10], *p* = 0.007). Parental asthma management self-efficacy was significantly related to family functioning (β = 0.11, 95% CI [0.09, 0.25], *p* = < 0.001); however, its relation to psychological adjustment was not significant (*p* = 0.116). The predictors accounted for 62% of the variance in family functioning and 58% of the variance in psychological adjustment, respectively.

**Figure 2:**
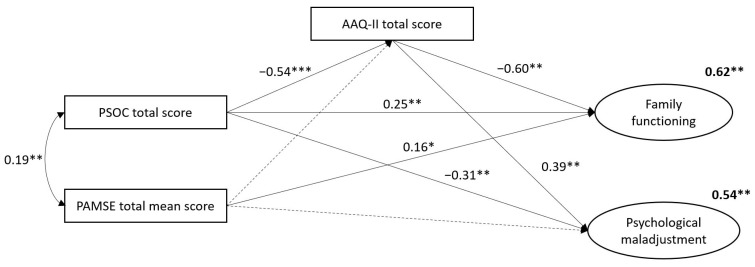
Mediation analysis. Note. Latent variables are presented as ellipses, and observed variables are presented as rectangles. Solid lines illustrate statistically significant relationships, and dashed lines illustrate those that are not. Squared multiple correlation coefficients, showing the variance that each variable explains, are placed in the upper right corner of each variable’s representation. The model was adjusted for the following sociodemographic variables: parents’ age and relationship with the child, the child’s age and sex, and the current use of inhaled corticosteroids and CNS and/or non-CNS stimulants for asthma and ADHD symptom management. Insignificant confounders and their associated paths were subsequently removed to streamline the model. AAQ-II, Acceptance and Action Questionnaire-II; PSOC, Parenting Sense of Competency; PAMSE, Parents’ Asthma Management Self-Efficacy; FIM, Pediatric Quality of Life Inventory Family Impact Module; and PECI, Parent Experience of Child Illness. Significance is indicated with * *p* < 0.05, ** *p* < 0.01, and *** *p* < 0.001.

Figure 2 presents the full trimmed mediation analysis examining the mediating role of psychological flexibility. The goodness-of-fit indices indicated an acceptable model fit (χ²/df = 129.57/81 = 1.59, RMSEA = 0.11, CFI = 0.91, TLI = 0.89, SRMR = 0.07). The Bollen–Stine bootstrap test yielded a *p*-value of 0.11, indicating a lack of statistical significance. Psychological inflexibility was found to partially mediate the relationship between (1) parenting competence and family functioning (indirect effect: β = 0.32, 95% CI [0.19, 0.50], *p* = < 0.001, proportion mediated = 0.56) and (2) parenting competence and psychological adjustment (indirect effect: β = −0.21, 95% CI [−0.34, −0.10], *p* = < 0.001, proportion mediated = 0.41). However, psychological inflexibility did not mediate the effect of parental asthma management self-efficacy on family functioning (*p* = 0.61) or on psychological adjustment (*p* = 0.14).

## 4. Discussion

The present study aimed to investigate whether asthma management self-efficacy, parenting competence, and psychological flexibility (PF) simultaneously influence psychological adjustment and family functioning in the parents of children diagnosed with asthma and comorbid ADHD. Additionally, the study examined whether psychological flexibility mediated these associations. This integrative approach reflects an effort to capture the complexity of parental experiences when raising a child with comorbid conditions, as well as the multifactorial influences on family functioning and psychological adjustment.

Our findings highlight the dual associations of PF with both family functioning and psychological adjustment, while accounting for intercorrelations with parenting competence and asthma management self-efficacy. As a construct encompassing openness, awareness, and committed action, PF empowers parents to respond to stress with adaptive coping strategies [55]. The current literature supports the link between elevated PF and enhanced family functioning [41,56,57], with such parents not only employing effective parenting strategies but also demonstrating improved communication, conflict resolution, emotional support, and overall family cohesion. Furthermore, PF is instrumental in parental psychological adjustment, providing a buffer against negative cognitive and emotional states and protecting against mental health challenges [58,59]. Research has consistently shown that greater PF is associated with improved mental health outcomes, including fewer symptoms of depression and anxiety [60,61,62]. Furthermore, PF has been identified as a mediator that can attenuate mental health issues among parents of children with an array of health conditions, including asthma [63,64], cancer [65], life-threatening illnesses [66], and neurodevelopmental disorders [67]. While the aforementioned studies focused on the role of parental PF among families with children experiencing a single-condition chronic illness, our research extends this to comorbid conditions, a less explored area. Our evidence suggests that enhancing parental PF is vital in supporting psychological adjustment and family functioning amidst the complex challenges of comorbidity.

We also examined the association between parental self-efficacy—the belief in one’s ability to manage parenting responsibilities—and family functioning and psychological adjustment. Contrary to our hypothesis, no significant relationship was found between parental self-efficacy and these outcomes. This finding is noteworthy when considered alongside the substantial associations identified for PF. While self-efficacy is undoubtedly important in managing specific parenting tasks, our results suggest that it may not play as pivotal a role in the broader context of family dynamics as PF does. Unlike the task-specific confidence involved in managing particular situations, PF represents a broader capacity to navigate the emotional and cognitive challenges of parenting, particularly when caregiving involves children with comorbid chronic conditions [36,68]. PF enables parents to remain adaptable, align their actions with deeply held values, and respond to fluctuating demands with psychological resilience. This flexibility allows for a more stable and value-driven approach to parenting, fostering improved family functioning and psychological adjustment more consistently than parenting competence alone. Moreover, the mediating role of PF in the relationship between parenting competence and both family functioning and psychological adjustment—but not in the relationship between parental asthma management self-efficacy and these outcomes—highlights the different roles of these constructs. While asthma management self-efficacy is essential for addressing specific health needs, it does not engage the broader emotional and cognitive flexibility that PF facilitates. By promoting emotional agility and the ability to cope with stressors across various life domains, PF supports a more holistic approach to parental well-being and adaptive functioning [39,68]. This generalizability across contexts explains why PF serves as a more foundational mechanism in promoting adaptive family dynamics and psychological adjustment, particularly in the context of complex parenting challenges.

### Study Limitations and Implications

Our findings have provided deeper insights into the roles of psychological flexibility, parenting competence, and disease management self-efficacy in parents managing their children’s asthma when it is comorbid with ADHD. This complex scenario presents significant challenges, as parents must navigate the demands of a chronic medical condition alongside their child’s behavioral and emotional difficulties. The higher frequency of unscheduled medical visits and hospitalizations observed in our sample (30%), compared to a similar local study in parents of children with asthma (<10%) [63], may be influenced by comorbid ADHD, which can complicate asthma management through difficulties in adherence. ADHD-related impairments in attention and executive functioning could lead to inconsistent medication use [23] and thereby increase the risk of asthma exacerbations.

While the research has provided valuable insights, it is not without limitations. The cross-sectional design limits the establishment of causal and directional relationships. The measurement of PF using the Acceptance and Action Questionnaire-II (AAQ-II) is subject to scrutiny regarding its discriminant validity and its potential conflation with psychological distress [69]. Moreover, the over-representation of mothers in the sample may constrain the extrapolation of findings to fathers or other caregivers. In light of these findings, future research should adopt longitudinal methods and cross-lagged models to better ascertain the causality and directionality of the observed relationships. A gender-balanced sample would improve the findings’ generalizability. The incorporation of alternative PF measures, such as the Parental Psychological Flexibility Questionnaire [70], the 6-Parental Acceptance Questionnaire [71], and the Parenting Specific Psychological Flexibility Scale [68], which had been validated within the context of parents caring for children with chronic conditions [72], may alleviate measurement concerns.

Despite these limitations, this study’s findings offer valuable implications for interventions aimed at improving parental psychological adjustment and family functioning. The strong associations observed between psychological flexibility (PF) and both family functioning and psychological adjustment underscore PF as a critical target for intervention. Programs grounded in Acceptance and Commitment Therapy (ACT) [73], which aim to enhance PF, could lead to significant improvements in parenting competence and parental self-efficacy. By enabling parents to manage their thoughts and emotions more effectively, these interventions could foster adaptability and resilience, thereby enhancing their ability to execute caregiving tasks with greater competence and confidence.

While parenting competence and self-efficacy in disease management are important, our findings advocate for prioritizing PF as the central component of interventions aimed at improving individual and family well-being. Although the severity of a child’s asthma and/or ADHD can challenge and potentially diminish parental PF due to the demands of managing impulsivity, inattention, and emotional dysregulation [74], our findings highlight that parents who maintain greater PF are better equipped to positively influence family functioning and psychological adjustment. This suggests that even in the face of significant stressors like ADHD, the cultivation of PF in parents can serve as a protective factor, enabling more effective coping and promoting overall family resilience. Psychological flexibility thus equips parents with the resilience needed to navigate the complex challenges posed by their children’s comorbid conditions. Integrating ACT into existing asthma management programs or positive parenting programs could therefore provide more holistic support, ultimately strengthening the entire family system. Researchers and clinicians should consider these implications when designing supportive interventions, with PF emphasized as a key element in the comprehensive management of pediatric comorbidity.

## 5. Conclusions

Our analysis reveals the influential role of parental PF and parenting competence in promoting psychological adjustment and optimal family functioning, extending beyond the effects of self-efficacy. By fostering parental PF through ACT and reinforcing behavioral competence in parenting via established programs like the Positive Parenting Program, there is potential for marked enhancements in family interactions and the psychological well-being of parents. This goes beyond the conventional focus on bolstering parental confidence solely for managing symptoms. Adopting this broad approach to intervention strategies is expected to make a significant and meaningful contribution in the realms of pediatric chronic illness management and the provision of parental support.

## Figures and Tables

**Table 1:** Zero-order correlation matrix of the study variables.

	Variable, Correlation													
Variable (No.)	1	2	3	4	5	6	7	8	9	10	11	12	13	14
AAQ2 total score (1)	1													
PAMSE total mean score (2)	−0.11 *	1												
PSOC total score (3)	−0.53 **	0.21 *	1											
PECI—Guilt and worry (4)	0.55 **	−0.10	−0.42 **	1										
PECI—Unresolved sorrow and anger (5)	0.42 **	−0.15	−0.41 **	0.67 **	1									
PECI—Long-term uncertainty (6)	0.55 **	−0.18 *	−0.5 3**	0.71 **	0.60 **	1								
FIM—Physical functioning (7)	−0.39 **	0.25 **	0.26 **	−0.26 **	−0.30 **	−0.33 **	1							
FIM—Emotional functioning (8)	−0.63 **	0.07	0.43 **	−0.48 **	−0.42 **	−0.48 **	0.53 **	1						
FIM—Social functioning (9)	−0.42 **	0.15	0.37 **	−0.36 **	−0.37 **	−0.41 **	0.49 **	0.64 **	1					
FIM—Cognitive functioning (10)	−0.45 **	0.12	0.31 **	−0.30 **	−0.28 **	−0.35 **	0.41 **	0.45 **	0.34 **	1				
FIM—Communication functioning (11)	−0.45 **	0.20 *	0.38 **	−0.33 **	−0.41 **	−0.52 **	0.42 **	0.49 **	0.56 **	0.52 **	1			
FIM—Worry (12)	−0.49 **	0.19 *	0.37 **	−0.52 **	−0.50 **	−0.68 **	0.39 **	0.56 **	0.37 **	0.38 **	0.44 **	1		
FIM—Daily activities (13)	−0.50 **	0.16	0.32 **	−0.32 **	−0.35 **	−0.41 **	0.45 **	0.52 **	0.38 **	0.55 **	0.51 **	0.48 **	1	
FIM—Family relationship (14)	−0.49 **	0.14	0.36 **	−0.19 *	−0.34 **	−0.34 **	0.40 **	0.39 **	0.41 **	0.45 **	0.47 **	0.45 **	0.58 **	1

Note. AAQ-II, Acceptance and Action Questionnaire-II; PECI, Parent Experience of Child Illness; PAMSE, Parent Asthma Management Self-Efficacy; PSOC, Parenting Sense of Competency Scale; FIM, Pediatric Quality of Life Inventory Family Impact Module. * *p* < 0.05; ** *p* < 0.01.

## Data Availability

The study reported in this article was not formally preregistered. Neither the data nor the materials have been made available in a permanent third-party archive; requests for the data or materials can be sent via email to the lead author at conniechong@cuhk.edu.hk.

## Appendix A

**Table A1:** Confirmatory factor analysis of the latent constructs.

Latent Constructs/Indicators	Confirmatory Factor Analysis	
Psychological Adjustment	Standardized Factor Loading	*p* Value
PECI—Guilt and worry	0.85	<0.001
PECI—Unresolved sorry and anger	0.74	<0.001
PECI—Long-term uncertainty	0.74	<0.001
Family functioning		
FIM—Physical functioning	0.63	<0.001
FIM—Emotional functioning	0.88	<0.001
FIM—Social functioning	0.71	<0.001
FIM—Cognitive functioning	0.70	<0.001
FIM—Communication functioning	0.86	<0.001
FIM—Worry	0.70	<0.001
FIM—Daily activities	0.74	<0.001
FIM—Family relationship	0.71	<0.001

Note. PECI, Parent Experience of Child Illness; PSOC, Parenting Sense of Competency Scale; FIM, Pediatric Quality of Life Inventory Family Impact Module.
